# Mechanisms of RNA N^6^-Methyladenosine in Hepatocellular Carcinoma: From the Perspectives of Etiology

**DOI:** 10.3389/fonc.2020.01105

**Published:** 2020-07-07

**Authors:** Jiahua Lu, Junjie Qian, Shengyong Yin, Lin Zhou, Shusen Zheng, Wu Zhang

**Affiliations:** ^1^Division of Hepatobiliary and Pancreatic Surgery, Department of Surgery, First Affiliated Hospital, School of Medicine, Zhejiang University, Hangzhou, China; ^2^Key Laboratory of Combined Multi-Organ Transplantation, Ministry of Public Health, Hangzhou, China; ^3^Key Laboratory of the Diagnosis and Treatment of Organ Transplantation, CAMS, Hangzhou, China; ^4^Collaborative Innovation Center for Diagnosis Treatment of Infectious Diseases, Zhejiang University, Hangzhou, China; ^5^Shulan (Hangzhou) Hospital Affiliated to Zhejiang Shuren University Shulan International Medical College, Hangzhou, China; ^6^Institution of Organ Transplantation, Zhejiang University, Hangzhou, China

**Keywords:** m^6^A methylation, hepatocellular carcinoma, molecular mechanism, cancer etiology, viral hepatitis, non-alcohol fatty liver disease

## Abstract

N6-Methyladenosine (m^6^A) is the most common RNA internal modification in eukaryotic cells. Its regulatory effects at the post-transcriptional level on both messenger RNAs (mRNAs) and noncoding RNAs have been widely studied; these include alternative splicing, stability, translation efficiency, nucleus export, and degradation. m^6^A modification is implicated in a series of physiological and pathological activities, such as embryonic stem cell differentiation, immunoregulation, adipogenesis, and cancer development. Recently, the significance of m^6^A methylation has been identified in both viral hepatitis and non-alcohol fatty liver disease (NAFLD), which are major risk factors in the development of hepatocellular carcinoma (HCC). Given the high incidence and mortality rate of HCC worldwide, it is of great importance to elucidate the mechanisms underlying HCC initiation and progression. m^6^A as an emerging research focus has great potential to facilitate the understanding of HCC, particularly from an etiological perspective. Thus, in this review, we summarize recent progress in understanding m^6^A modification related to viral hepatitis, NAFLD, and HCC, including their mechanisms and clinical applications.

## Introduction

Hepatocellular carcinoma (HCC) is the fourth leading cause of cancer-related death worldwide and accounts for more than 80% of primary liver cancers (1). Viral hepatitis and non-alcoholic fatty liver disease (NAFLD) are two significant risk factors for HCC development (2–4). The infection rates of hepatitis B virus (HBV) and hepatitis C virus (HCV) remain significant in high-risk areas, although vaccines and effective medicines have been designed for the prevention and treatment of viral hepatitis (5, 6). With the global epidemic of obesity, NAFLD has emerged as another nonnegligible force in HCC etiology (7). Due to asymptomatic disease progression, most patients have already advanced into liver cirrhosis or even HCC at the first diagnosis (3). Hence, these patients are not eligible for curative treatments such as surgical resection or liver transplantation, and instead are left with the exclusive choice of palliative therapies. Worse still, first-line-medicines such as sorafenib can only extend the overall survival of patients for another 3 months, and the response rate of the emerging programmed cell death ligand-1 (PD-L1) immune checkpoint inhibitor is lower than 20% in HCC patients (8, 9). Thus, it is of great urgency to develop novel therapies for the effective prevention, early diagnosis and precision treatment of HCC.

N^6^-methyladenosine (m^6^A) is a ubiquitous RNA internal modification at the posttranscriptional level; it was first identified in eukaryotic cells, and later found in prokaryotic cells and viruses (10–15). In 2012, the landscape of m^6^A modification was for the first time identified at the whole-transcriptome level (16, 17). Most m^6^A sites are enriched within a consensus motif of RRACH (R = G or A, A = m^6^A and H = A, C, or U), and are preferentially located around stop codons and within long internal exons. Regulators of m^6^A modification include “writers,” which are methyltransferases responsible for transferring the methyl group to the N6 position; “readers,” which are RNA-binding proteins that recognize specific m^6^A-modified positions to regulate RNA functions; and “erasers,” which are demethylases that mediate this reversible biological process (18–20).

m^6^A modification regulates the metabolic processes of both messenger RNAs (mRNAs) and noncoding RNAs (ncRNAs), which include structural stability, alternative splicing, translation efficacy, export, and decay (21–24). The reciprocal effects of m^6^A methylation with mRNAs or ncRNAs are associated with a series of physiological and pathological biological behaviors such as stem cell differentiation, immunoregulation and carcinogenesis (25–27). There is mounting evidence that m^6^A dysregulation is critically involved in HCC occurrence and development. For example, elevated m^6^A levels in HCC fuel inflammation and neovascularization in tumors by inhibiting m^6^A readers (28). In addition, a reduction of m^6^A modification promotes HCC metastasis in an m^6^A-dependent manner by modulating microRNA (miRNA) processing (29). Furthermore, some m^6^A regulators have shown clinical value as biomarkers or therapeutic targets in HCC (30). Therefore, in this review, we mainly summarize the potential mechanisms of m^6^A modification in HCC development from an etiological perspective, as well as its possible clinical applications, including as biomarkers and therapeutic targets.

### Regulators of m^6^A Modification

**Figure 1:**
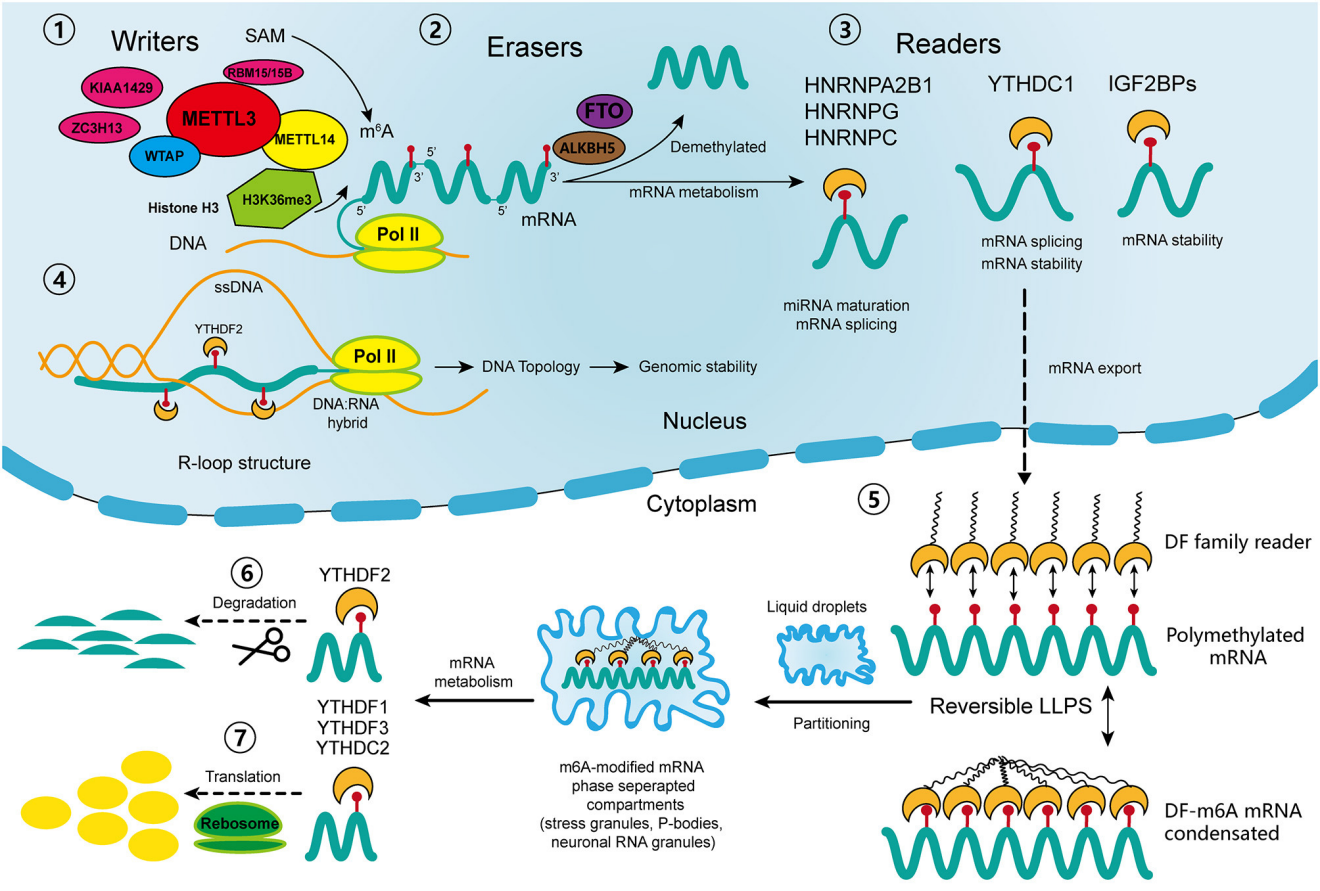
Schematic diagram of the process and biological function of RNA m^6^A methylation. The methyltransferase complex composed of METTL3, METTL14 and WTAP co-transcriptionally catalyzes the transfer of the methyl group from adenosylmethionine (SAM) onto the N6 position of adenosine; other “writers” include RBM15/15B, KIAA1429, and ZC3H13. With the guidance of histone H3K36me, the m6A sites are preferentially located near the 3′ terminus of the transcripts. Demethylases FTO and ALKBH5 reverse the m^6^A process. In nucleus, the “readers” of YTHDC1, IGF2BPs, HNRNPs participate in diverse RNA biological processes, including mRNA alternative splicing, stability, export, and miRNA maturation. The m^6^A-modified R-loop structures are implicated in the regulation of genomic stability. In cytoplasm, the m6A-modified mRNAs are targeted for regulation through a process called liquid-liquid phase separation (LLPS), which is mediated by YTHDFs. Then, other “readers” regulate the degradation and translation of mRNAs in the cytoplasm. ssDNA, single strand DNA; pol II: RNA polymerase II.

The m^6^A regulators, including “writers,” “erasers,” and “readers,” cooperatively maintain the dynamic and reversible balance of m^6^A methylation (31) (Figure 1). “Writers” including methyltransferase-like 3 (METTL3), METTL14, and Wilm's tumor 1-associated protein (WTAP) comprise the major components of the methyltransferase complex within the nucleus, which is responsible for the m^6^A methylation process (20). METTL3 is the core catalytic enzyme for transferring methyl groups to N^6^ positions, while METTL14 is vital for recognizing and stabilizing the METTL3-METTL14 complex (32). WTAP binds the methyltransferase complex and recruits it toward mRNA targets (33). Other identified “writers” include METTL16, KIAA1429, zinc finger CCCH domain-containing protein 13 (ZC3H13), and RNA-binding motif protein 15 (RBM15) (20). It was not until 2011 that scientists discovered that m^6^A modification could be reverted by demethylase, which drew the attention of the broader academic community (34). Then it was discovered that the dynamic demethylation process could be achieved by “erasers” including AlkB homolog 5 (ALKBH5), and fat mass and obesity-associated protein (FTO). *FTO* was the first identified gene contributing to human obesity, and its polymorphisms are closely related to insulin resistance and metabolic diseases (35, 36). Two studies reported the identical increase in intracellular m^6^A methylation after knocking out two different “erasers” (34, 37). Moreover, the demethylation of FTO and ALKBH5 mainly affects m^6^A residues within the nucleus.

The most studied m^6^A “readers” are YTH domain-containing proteins, including YTHDF1-3 (located in the cytoplasm), YTHDC1 (in the nucleus), and YTHDC2 (in both) (19). YTHDF1 and YTHDF3 have coordinated functions that promote the translation of m^6^A-modified mRNAs, while YTHDF2 expedites mRNA degradation, and YTHDC1 takes part in mRNA alternative splicing and nuclear export (38–40). YTHDC2 acts paradoxically in either promoting translation or accelerating mRNA degradation (41). Recently, a novel cellular biological process called liquid-liquid phase separation (LLPS) was discovered, through which degradation, translation and splicing of m^6^A-modified mRNAs are regulated (42). Some low-complexity domains belonging to YTHDFs have the ability to interact with each other and to partition into liquid droplets within the cytoplasm (43). This process can be greatly enhanced by mRNA transcripts containing multiple m^6^A residues, which recruit and juxtapose YTHDF proteins, initiating the phase separation process. Subsequently, the mRNA-YTHDF complexes partition into endogenous phase-separated liquid droplets, such as stress granules, P-bodies, or neuronal RNA granules, thereby transforming into membraneless compartments, where their degradation, splicing, and transportation are regulated (42). In addition to the YTH reader proteins, there are some binding proteins regulated by m^6^A-induced structural changes called m^6^A “switches,” which include heterogeneous nuclear ribonucleoprotein C (HNRNPC), HNRNPG, HNRNPA2B1, and insulin-like growth factor 2 mRNA binding protein 1-3 (IGF2BP1-3) (44). HNRNPC and HNRNPG are involved in mRNA splicing, and HNRNPA2B1 participates in miRNA maturation (18). IGF2BPs function through the recruitment of RNA stabilizers, such as Hu antigen R (HuR), an RNA stabilizer, to maintain mRNA stability (45–47). However, the detailed mechanisms of their interactions with m^6^A-modified sites require further exploration.

In addition to the well-studied functions of m^6^A regulators, there are some newly identified mechanisms that participate in the regulation of m^6^A modification. Huang et al. discovered that histone H3 trimethylation at Lys36 (H3K36me3), one of the transcriptional markers, interacts with METTL14 to guide the methyltransferase complex to nascent RNA and to specific regions of mRNA transcripts (48). In that study, silence of either METTL14 or H3K36me greatly reduced the m^6^A levels throughout the transcriptome. Moreover, the overlapped sites of histone modification and m^6^A modification are preferentially located near the coding sequence (CDS) and 3′ terminus of the transcripts. This finding partially explained the site specificity of m^6^A modification in mRNA transcripts, and revealed another layer of regulatory mechanism involving crosstalk between m^6^A methylation and histone modification. Other recent studies have shown that m^6^A modification regulates not only the stability of mRNAs but also DNA structures. R-loops are nucleic acid structures consisting of three strands: an RNA: DNA hybrid and a single strand of unpaired DNA (49). They actively regulate genome dynamics and functions, including immunoglobulin class switching, transcription initiation and termination as well as genomic stability (49). Two independent studies have confirmed the existence of m^6^A modified-RNAs within R-loops. Abakir et al. found that YTHDF2 recognized m^6^A-modified R-loops and promoted their degradation (50). By contrast, Yang et al. observed a decreased R-loop levels upon METTL3 silencing, which suggests that m^6^A may promote R-loop formation (51). Although there are discrepancies between these two studies, they both indicate that m^6^A plays an important role in maintaining genomic integrity by modulating the accumulation of R-loops. Further, this modulation prevented the occurrence of a variety of diseases such as cancers, Kaposi's sarcoma and neurological disorders. In addition, Liu et al. identified METTL3-catalyzed m^6^A on chromosome-associated regulatory RNAs (carRNAs), such as enhancer RNAs, repeats RNAs and promoter-associated RNAs, and YTHDC1 promotes the degradation of these RNAs (52). Moreover, a reduction of m^6^A modification increases carRNA levels and promotes the open chromatin state and transcription. This finding suggests a regulatory effect of m^6^A on carRNAs, which in turn affects the chromatin state and transcription.

### Role of m^6^A Modification in Hepatocellular Carcinoma

**Figure 2:**
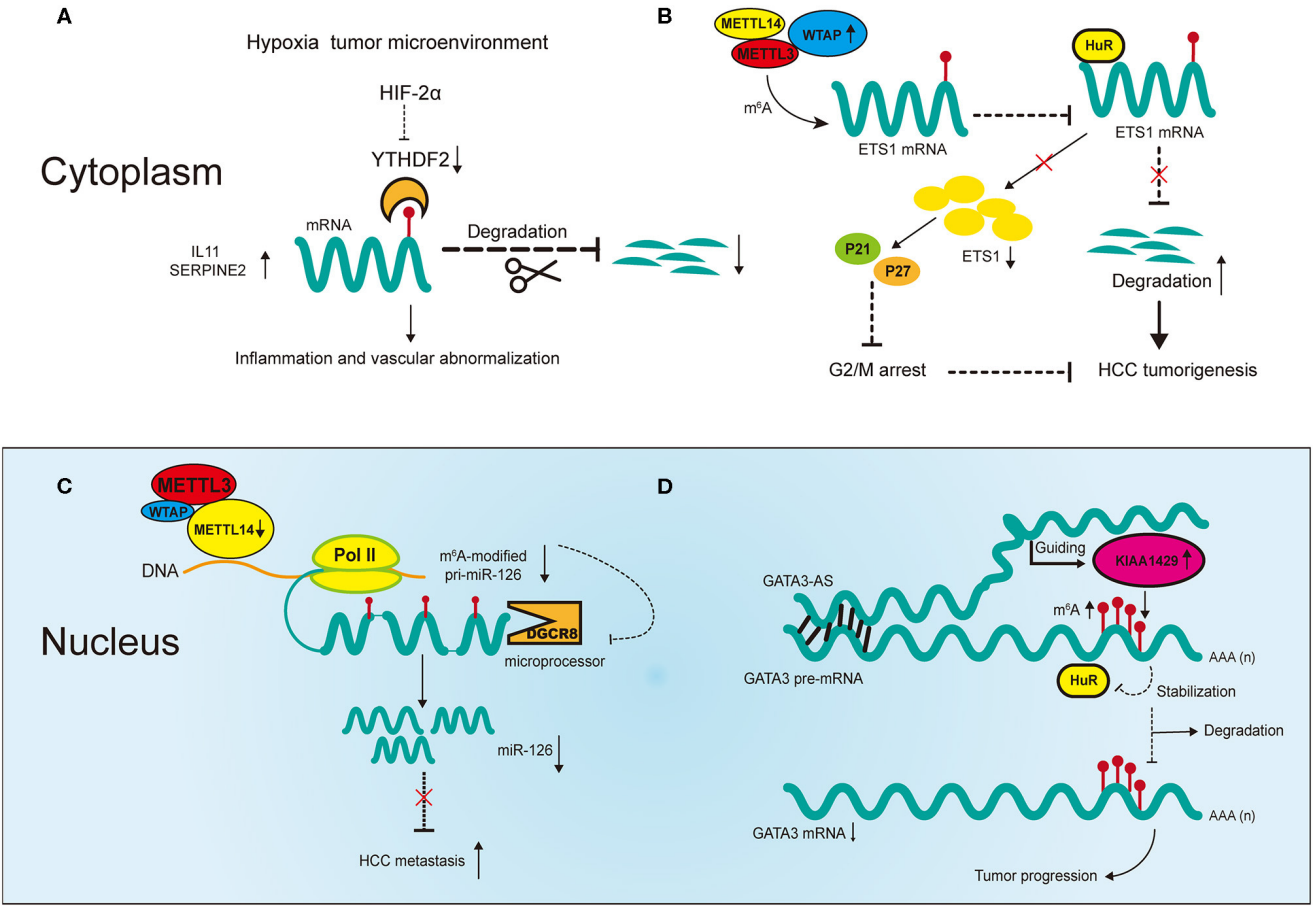
Partial m^6^A regulatory mechanisms involved in messenger RNAs (mRNAs) and noncoding RNAs (ncRNAs) in HCC. **(A)** The hypoxia tumor microenvironment negatively regulates YTHDF2 to promote cancer inflammation and vascular abnormalization. **(B)** Upregulated WTAP promotes HCC tumorigenesis by destabilizing its targeted mRNAs in an m^6^A-dependent manner. **(C)** Downregulated METTL14 promotes HCC metastasis by inhibiting microRNA 126 (miRNA) maturation in an m^6^A-dependent manner. **(D)** Upregulated KIAA1429 promotes HCC progression in an m^6^A- and lncRNA-dependent manner.

The regulators of m^6^A play a pleiotropic role in the modulation of HCC, and both mRNA and ncRNA participate in m^6^A-mediated biological processes in HCC (Figure 2). The m^6^A regulators reviewed in this article include the “writers” METTL3, METTL14, WTAP, and KIAA1429; the “eraser” FTO and the “readers” YTHDF1, YTHDF2 and IGF2BP1; their regulatory effects in HCC are summarized in Table 1.

**Table 1:** Role of m6A regulators in HCC occurrence and development.

**Diseases**	**Regulators**	**Specific function**	**Mechanisms**	**References**
HBV	METTL3	Promoting viral replication and inhibiting viral proteins expression	Promoting reverse transcription and reducing HBV RNA stability	(81)
	METTL14			
	YTHDF2			
	YTHDF3			
HCV	YTHDF	Inhibiting viral infection	Inhibiting infectious virions production by relocating them to lipid droplets where virions assemble	(79)
	METTL3			
	METTL14			
NAFLD	FTO	Promoting adipogenesis and enhancing adipocyte differentiation	Promoting alternative splicing of RUNX1T1 by inhibiting RNA-binding of SRSF2	(87)
	FTO	Promoting adipogenesis	Inhibiting YTHDF2-mediated degradation of CCNA2 and CDK2	(108)
	FTO, METTL3		FTO promoting lipid accumulation while METTL3 inhibiting adipogenesis	(109)
	METTL3	Facilitating insulin resistance and accelerating fatty acid metabolism *in vitro*	Promoting Fasn expression	(84)
	METTL14	Promoting insulin resistance, lipogenesis and lipolysis *in vivo*	Activating the AKT signaling	(83)
HCC	METTL3	Promoting proliferation, migration and colony formation *in vitro*, promoting tumorigenesis and metastasis *in vivo*	Inhibiting SOCS2 expression by facilitating YTHDF2-mediated SOCS2 degradation	(65)
		METTL3-mediated upregulation of LINC00958 promoting proliferation, migration and lipogenesis in HCC cells	Upregulating LINC00958 by enhancing its stability	(72)
		Promoting EMT phenotypes *in vitro* and *in vivo*	Promoting YTHDF1-mediated translation of Snail mRNA	(53)
	WTAP	Promoting proliferation and tumor growth *in vitro* and *in vivo*	Inhibiting ETS1 expression by destabilizing its binding with HuR	(66)
	METTL14	Inhibiting HCC metastasis	Promoting pri-miR-126 processing by binding to DGCR8	(29)
	KIAA1429	Inhibiting proliferation and metastasis *in vitro* and *in vivo*	Promoting GATA3 pre-mRNA decay by inhibiting HuR	(73)
		Promoting HCC cells migration and invasion	Inhibiting ID2	(68)
	YTHDF2	Inhibiting hypoxia-induced inflammation, angiogenesis and metastasis in HCC	Inhibiting IL11 and SERPINE2 by promoting their degradation	(28)
		Suppressing proliferation and tumor growth	Promoting the decay of EGFR mRNA by binding to its 3′ terminus	(64)
		Positively associated with HCC malignancy	Inhibited by miR-145 binding to its 3′ terminus	(71)
	IGF2BP1	Promoting cancer cell growth and invasion	Inhibiting miRNA-mediated degradation of SRF transcripts	(67)
	ZCCHC4	Promoting proliferation *in vitro* and tumor growth *in vivo*	Promoting 28S ribosomal RNA methylation	(75)
	FTO	Promoting proliferation and *in vivo* tumor growth	Increasing PKM2 expression	(69)
	METTL3, YTHDF1	Predicts worse survival	Overexpressed in HCC tissues	(76, 77)
	METTL14	Predicts worse survival	Regulating CSAD, GOT2 and SOCS2	(30)

#### m^6^A in Hepatocellular Carcinoma and Other Cancers

Dysregulation of m^6^A methylation and abnormal expression of regulators are involved in various cancer functions, such as distant metastasis, cancer immunoregulation, tumor angiogenesis and cancer stem cell formation, by modulating the degradation, processing or translation of the downstream targeted RNAs. The epithelial mesenchymal transition (EMT), a critical process for cancer cell metastasis, is regulated by the m^6^A writer METTL3, not only in HCC but across diverse cancers. Lin et al. discovered that METTL3 is upregulated in HCC, promoting EMT by enhancing the m^6^A modification of Snail mRNA (53). Moreover, YTHDF1 mediates the m^6^A-increased translation of Snail mRNA, which promotes the HCC metastasis. Notably, METTL3 also participates in EMT in gastric cancer (GC), ovarian cancer and prostatic cancer (27, 54). Yue et al. found that m^6^A modification of zinc finger MYM-type containing 1 (ZMYM1), a downstream target of METTL3, enhanced its stability through the regulation of HuR, and that ZMYM1 represses E-cadherin promoter by recruiting the CtBP/LSD1/CoREST complex, thereby facilitating the EMT process and metastasis of GC (55). Intriguingly, Ma et al. reported that METTL14 suppresses HCC metastasis by regulating miRNA in an m^6^A-dependent manner (29). METTL14 also inhibits the progression and metastasis of colorectal cancer (CRC) by downregulating oncogenic lncRNA (56). Moreover, it can inhibit CRC cell growth via miR-375 /Yes-associated protein 1 (*YAP1*) pathway (57). These findings confirm the significant roles of m^6^A writers in regulating tumor metastasis and progression across diverse cancers.

It is noteworthy that, because m^6^A modification and the expression of m^6^A regulators are highly heterogeneous across various cancer types, the effects of m^6^A methylation may differ even in the same context. For instance, hypoxia in breast cancer stimulates hypoxia-inducible factor (HIF)-1α and HIF-2α- dependent expression of ALKBH5, which leads to the demethylation of NANOG mRNA and the induction of cancer stem cell phenotype (58). In non-small cell lung cancer (NSCLC), YTHDF1 is identified as a hypoxia adaptor, whose depletion inhibits tumor progression by regulating translation efficacy (59). However, in HCC, hypoxia induces the reduction of YTHDF2, which promoted tumor inflammation and angiogenesis (28). Additionally, YTHDF2 is recognized as an oncogene that is upregulated in lung cancer and acute myeloid leukemia (AML) to promote tumor initiation and growth, exhibiting a completely opposite function as in HCC (60, 61).

The functional implications of m^6^A in tumor immunology are drawing increasing attention. Yang et al. reported overexpression of FTO in melanoma, and that its knockdown increased the m^6^A level of critical tumorigenic genes such as *PD-1, CXCR4*, and *SOX10*, leading to increased mRNA degradation mediated by YTHDF2 (62). Moreover, silencing of FTO sensitized melanoma to anti-PD-1 treatment. Li et al. found that overexpression of WTAP is associated with poor prognosis in GC patients, which might be correlated with T lymphocyte infiltration (63). However, to date, no studies have explored the relationship between m^6^A and immunoregulation in HCC. Due to the low response rate of advanced HCC patients to anti-PD-L1 treatment, m^6^A might be a promising new target for the study of drug insensitivity and resistance in immunotherapies.

Overall, the above evidence indicates that dysregulated m^6^A modification plays an important role in cancer biology, and this epigenetic alteration is highly heterogeneous.

#### m^6^A and mRNA in Hepatocellular Carcinoma

Two recent studies have revealed m^6^A mechanisms underlying the negative correlation between YTHDF2 and HCC under hypoxic conditions (28, 64). Hou et al. reported that reduced expression of YTHDF2 in HCC tumor tissues was accompanied by increases in m^6^A methylation and mRNA expression, and was associated with unfavorable survival outcomes and poor clinical classification (28). The study revealed that YTHDF2 inhibits HCC progression through m^6^A modification by decreasing the stability of interleukin 11 (*IL11*) and serpin family E member 2 (*SERPINE2*) mRNA, which are involved in tumor angiogenesis and inflammation activation respectively (Figure 2A). Moreover, repression of HCC by YTHDF2 was reversed by HIF-2α under hypoxic conditions. These findings reveal molecular mechanisms through which targeted HCC treatment may be utilized. Interestingly, Zhong et al. showed different mechanisms in HCC in terms of YTHDF2 reduction induced by hypoxia (64). YTHDF2 directly bound with the 3′ terminus of the EGFR m^6^A site to accelerate its decay, which impeded the MEK/ERK signaling and thereby suppressed HCC. In addition, the expression of YTHDF2 was repressed under hypoxic conditions in HCC. Although the study demonstrated the significant role of YTHDF2 in inhibiting HCC, whether it involves m^6^A modification requires validation in more experiments.

In addition to the direct regulatory effect of YTHDF2, there is evidence suggesting the cooperative function of m^6^A regulators in HCC. Chen et al. showed that METTL3 promotes HCC progression in a YTHDF2- and m^6^A-dependent manner (65). In that study, overexpression of METTL3 promoted tumor growth in *CRISPR/dCas9-VP64* models *in vivo* and *in vitro*, indicating its positive correlation with HCC. Suppressor of cytokine signaling 2 (*SOCS2*), a direct downstream target of METTL3, inhibited tumor progression. The expression of *SOCS2* was downregulated by METTL3 through m^6^A modification, in which YTHDF2 bound to the 3′ terminus of *SOCS2* to facilitate its decay. Therefore, the m^6^A regulators METTL3 and YTHDF2 cooperatively promoted the development of HCC in an m^6^A-dependent manner. Another regulator of m^6^A, WTAP, has also been found to promote HCC progression through m^6^A modification (Figure 2B) (66). WTAP is significantly upregulated in HCC and this is associated with unfavorable survival outcomes (66). ETS proto-oncogene 1 (*ETS1*), a tumor suppressor in HCC, is negatively regulated by WTAP via m^6^A modification. Moreover, m^6^A modification of ETS1 simultaneously inhibits its binding with HuR, the RNA stabilizer, which promotes its degradation. In addition, WTAP promotes the G2/M phase transition via ETS1-p21/p27 signaling pathway to regulate the HCC cell cycle. Overall, these results confirm the significance of cooperative effects between m^6^A regulators themselves and RNA stabilizers in HCC progression.

As m^6^A research advances, new regulators are being identified that take part in the biological processes of tumors. IGF2BPs (including IGF2BP1/2/3) are newly identified m^6^A reader proteins that promote the stability and storage of targeted transcripts by recognizing the consensus motif GGAC (A = m^6^A) (45). In HCC cells, IGF2BP1 upregulates serum response factor (*SRF*) by binding with its 3′ terminus and promoting its m^6^A modification (67). It also inhibits the miRNA-mediated degradation of *SRF* transcripts. The dual regulations enhance the expression of *SRF*-targeted genes thereby facilitating HCC tumorigenesis. Strikingly, there are 35 genes regulated by *SRF* or IGF2BP1 in total, which in combination construct an oncogenic cellular network in HCC, making themselves valuable therapeutic targets. Recently, KIAA1429 and FTO were both found to be overexpressed in HCC, which predicted unfavorable prognoses in HCC patients (68, 69). KIAA1429 inhibits the expression of inhibitor of DNA binding 2 (*ID2*) by promoting its m^6^A methylation while FTO downregulates pyruvate kinase M1/2 (*PKM2*) by triggering its demethylation. Consequently, KIAA1429 and FTO together promote the tumorigenesis of HCC. However, these results need higher levels of evidence for validation.

Overall, these findings suggest that mRNA stability and degradation are the most common m^6^A regulatory function in HCC. We believe that with more in-depth research on m^6^A regulators, new mechanisms will soon be revealed.

#### m^6^A and Noncoding RNAs in Hepatocellular Carcinoma

The regulatory effects of ncRNAs including long noncoding RNAs (lncRNAs), microRNAs and circular RNAs (circRNAs) on m^6^A are nonnegligible in HCC. Recently, several studies revealed how the interplay between m^6^A modification and ncRNAs influences the biological behaviors of HCC.

METTL14 inhibits HCC metastasis by regulating primary miR-126 (*pri-miR-126*) processing in an m^6^A-dependent manner (Figure 2C) (29). The processing of pri-miRNAs is usually performed by microprocessor proteins such as DiGeorge syndrome chromosomal region 8 (*DGCR8*) (70). In this study, overexpression of METTL14 upregulated the expression of miR-126 by promoting pri-miR-126 binding with DGCR8, which facilitated its processing. The same study found that in metastatic HCC, decreased METTL14 led to the accumulation of unprocessed pri-miR-126. Moreover, the inhibition of metastasis was reversed by miR-126 inhibitor in METTL14-overexpressing HCC cells, indicating the significance of miR-126 as a downstream target of METTL14 to inhibit metastatic HCC. In another study, miR-145 regulated m^6^A levels in HCC cells through the modulation of reader protein YTHDF2 (71). Overexpression of miR-145 increased m^6^A methylation in HCC cells and this regulation was reversed by overexpression of YTHDF2. Moreover, the luciferase activities confirmed that miR-145 directly targeted the 3′ terminus of YTHDF2 mRNA, and *in vitro* experiments proved its inhibition of HCC cell proliferation. This is the first report in HCC that ncRNAs regulate m^6^A regulators to influence cancer development. However, it should be noted that the effects of YTHDF2 in that study contradict earlier reports, which we suspected could be due to tumor heterogeneity in HCC (28, 64).

More recently, m^6^A modification has been reported to be correlated with the upregulation of LINC00958 in HCC, which may promote proliferation, invasion, migration and lipogenesis of HCC cells *in vitro* (72). Moreover, the upregulation of LINC00958 may be regulated by METTL3 via the modulation of the stability of RNA in HCC cells, and high expression of both genes may predict poor prognosis in HCC. However, this only shows that METTL3 is positively related with LINC0958, and thus more specific experimental methods are needed for validation. KIAA1429 can also promote HCC development via the m^6^A-dependent modification of GATA binding protein 3 (*GATA3*), in which its antisense transcript-derived lncRNA called GATA3-AS plays an important role (Figure 2D) (73). KIAA1429 is significantly upregulated in HCC and predicts worse survival outcomes in HCC patients. Knockdown of KIAA1429 is negatively related with hepatoma growth both *in vitro* and *in vivo*. KIAA1429 promotes the decay of *GATA3* precursor mRNA (pre-mRNA) through m^6^A modification, thereby promoting HCC progression. The RNA binding protein HuR increases the stability of *GATA3* pre-mRNA by binding with the 3′ terminus of pre-mRNA. However, this binding is negatively regulated by KIAA1429 through m^6^A modification. On the other hand, the lncRNA GATA3-AS acts as a cis-acting element for KIAA1429 to guide m^6^A methylation on GATA3 pre-mRNA 3′ terminus. This KIAA1429/GATA3 regulatory axis in HCC development reflects the breakdown of m6A dynamic balance under pathological conditions.

In recent research, circRNA has been found to facilitate HCC progression indirectly through m^6^A modification. CircRNA_104075 promotes *YAP*-related HCC progression via hepatocyte nuclear factor 4 α (*HNF4a*) in HCC (74). It also acts as a competitive endogenous RNA (ceRNA) to increase *YAP* expression by absorbing miR-583-3p. Intriguingly, an m^6^A motif, AGACU, has been identified within the 3′ terminus of *YAP*, which may be critical for the interplay between *YAP* and miR-582-3p. Furthermore, the critical effects of m^6^A was confirmed by luciferase activities and RNA immunoprecipitation quantitative polymerase chain reaction (RIP-qPCR) analyses. However, the mechanisms underlying this m^6^A modification should be further validated.

Overall, in HCC, m^6^A modification regulates ncRNAs in diverse ways, such as through splicing, preprocessing, stability and decay, and in turn ncRNAs affect and participate in the m^6^A process. Therefore, the interactions between m^6^A and ncRNAs play an important role in HCC progression, and we believe that more regulatory mechanisms will be identified in the future.

## Future Prospects and Clinical Applications of m^6^A in Hepatocellular Carcinoma

Considering the diverse biological effects mediated by m^6^A modification in HCC, there may ample opportunity to devise effective treatments and targeted therapies for HCC patients.

Recently, progress has been made on experimental therapies targeting m^6^A-specific mechanisms. Not only novel medicines but also new m^6^A regulators are being identified. Hou et al. demonstrated that PT2385 has excellent efficacy in the treatment of HCC, and significantly reduces tumor volume in subcutaneous HCC tumor models (28). It can reverse the reduction of YTHDF2 regulated by *HIF-2*α, which attenuates tumorous inflammation and angiogenesis thereby suppressing HCC growth. Although the administration of PT2385 does not affect the localization of YTHDF2 and cannot take effect with YTHDF2 deficiency, it does provide an opportunity to inhibit hypoxia-induced HCC development. Zuo et al. invented a novel PEGylated PLGA nanoplatform (PLGA-PEG NP) encapsulating si-LINC00958 targeting LINC00958 for the treatment of HCC (72). In the study, it exhibited controllable release, excellent cellular drug uptake and precise tumor-targeting capacity. Moreover, tumor size was effectively reduced in PLGA-PEG (si-LINC00958) NPs-treated HCC-patient-derived xenograft (PDX) model, and the pathological results and blood examination exhibited no significant treatment-related toxicity in the mouse model. A new m^6^A methyltransferase, zinc finger CCHC-type containing 4 (*ZCCHC4*), which mainly takes part in the methylation of 28S ribosomal RNAs (rRNAs), has been identified both *in vitro* and *in vivo* in HCC (75). Silence of ZCCHC4 decreases m^6^A methylation in 28S rRNA and inhibits proliferation of CRISPR/cas9-edited HepG2 cells. In addition, it is overexpressed in HCC tumor tissues and its silence significantly inhibits tumor growth in a xenograft animal model, confirming its significant role in promoting HCC progression through m^6^A methylation. Although its detailed biological functions in HCC require more validation, it might be a potential novel target for HCC treatment.

The application of m^6^A in HCC is not limited to clinical treatment; it may also be applied to cancer diagnosis and prognostic prediction. Recent studies have shown that m^6^A regulators have significant potential to serve as biomarkers. METTL3 and YTHDF1 are both overexpressed in HCC in several studies, and thus may be useful biomarkers for survival prediction and clinical classification (76, 77). Another study identified three METTL14-related downstream genes including cysteine sulfinic acid decarboxylase (*CSAD*), glutamic-oxaloacetic transaminase 2 (*GOT2*), and *SOCS2* using bioinformatics methods (30). They were included in a nomogram for the prediction of overall survival. Other overexpressed m^6^A regulators in HCC include WTAP, KIAA1429 and FTO, while downregulated enzymes include METTL14 and YTHDF2 (28, 66, 68, 69, 73).

## Relationship Between Hepatocellular Carcinoma and Viral Hepatitis and Non-alcoholic Fatty Liver Disease

Infection with hepatitis virus is the leading cause of HCC worldwide (3). There is a high rate of HBV infection in eastern Asia while HCV infection is more prevalent in developed countries (2, 5). The etiological connection between viral hepatitis and HCC enable us to intervene with the progression of disease for early prevention of HCC. HBV or HCV infection underlies the context of chronic liver inflammation, which would lead to liver cirrhosis and eventually HCC (3, 78). During the process of carcinogenesis, dysregulation of m^6^A may facilitate virus activity and disturb host immunity, causing persistent virus infection, shaping inflammatory microenvironment and consequently leading to HCC occurrence (Figure 2). For instance, depletion of certain m^6^A regulators in HCV can enhance viral activity, which increases virus titers without disturbing viral replication (79). Moreover, HCV infection affects host immunity in an m^6^A-dependent manner (80). m^6^A modification has also been observed in HBV, and is associated with virus reverse transcription (81). Thus, dysregulation of m^6^A in viral host cells is closely associated with the development of viral hepatitis, and may contribute to the occurrence of HCC.

NAFLD or non-alcohol steatohepatitis (NASH) is gaining increasing attention for its prevalence around the world and has become one of the leading etiologies in HCC (7). NAFLD is in fact a wide-range of chronic liver diseases, characterized by excessive accumulation of triglycerides in hepatocytes. It tends to progress from isolated hepatocyte triglyceride accumulation and steatosis (NAFL), to hepatic triglyceride accumulation with inflammation and hepatic injury (NASH), and eventually to liver fibrosis and HCC (82). NAFLD is strongly correlated with metabolic syndromes such as diabetes and obesity, and these risk factors interact to increase the risk for HCC occurrence in NAFLD patients. The pathogenesis of HCC in NAFLD patients is a complex process involving multiple mechanisms including lipogenesis, fat accumulation, insulin resistance, oxidative, and endoplasmic reticulum (ER) stress, inflammatory response and DNA damage (83–86) (Figure 2). Recent studies have indicated that m^6^A modification actively participates in these processes. The m^6^A demethylase FTO has been widely studied due to its established relationship with obesity, and it has been found to promote adipogenesis through multiple signaling pathways (87). Additionally, other m^6^A regulators such as METTL3 and METTL14 have been reported to participate in the regulation of insulin resistance (83, 84). These studies prove the significance of m^6^A modification in NAFLD progression, opening up an opportunity to prevent HCC in the future.

## Role of m^6^A Modification in Viral Hepatitis

**Figure 3:**
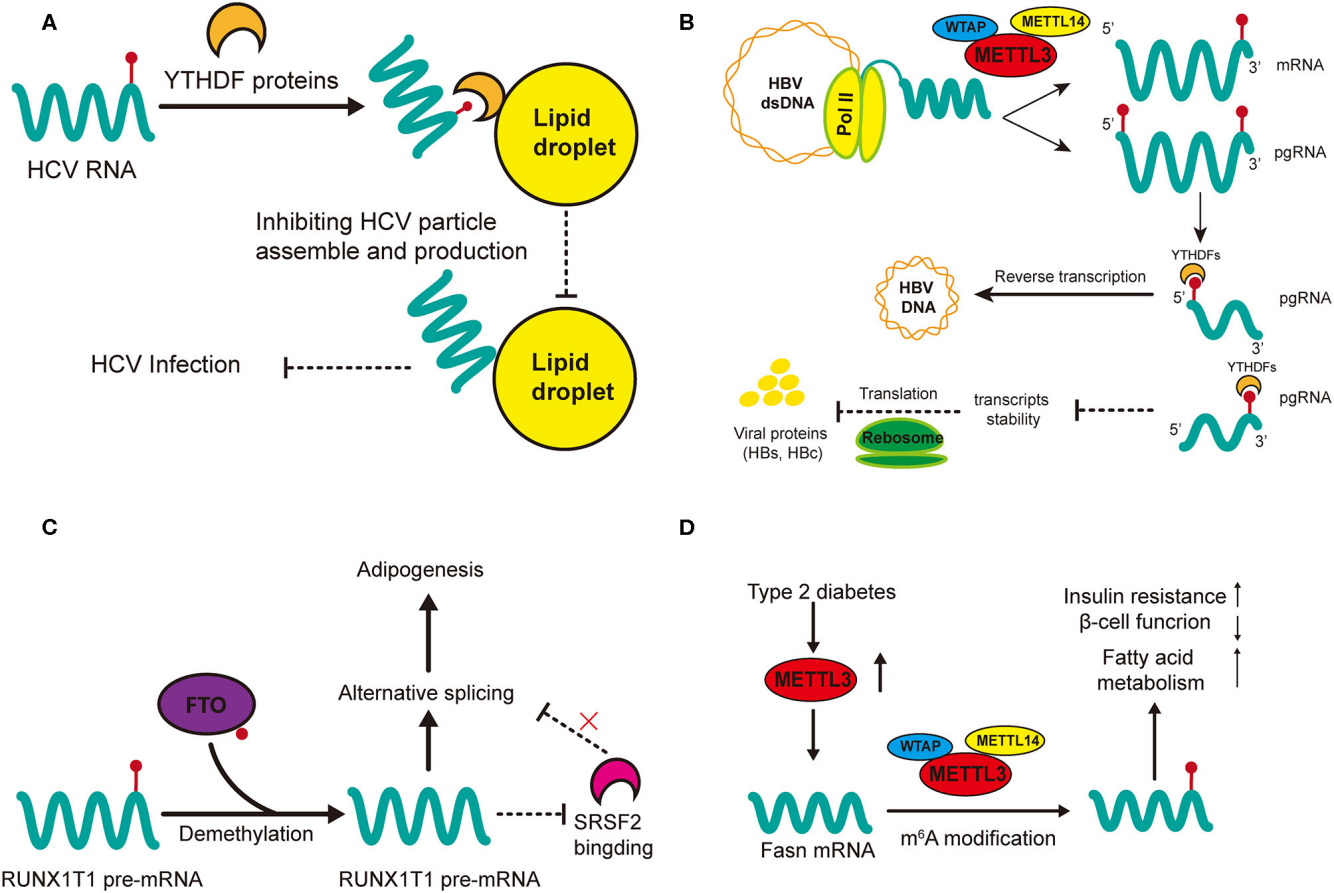
Regulatory functions of m^6^A in HCC-related hepatic diseases. m^6^A regulation in HCV **(A)** and HBV **(B)** affect viral activities (infection and replication) while m^6^A modification in non-alcohol fatty liver disease (NAFLD) promotes adipogenesis **(C)** and insulin resistance **(D)**. **(A)** m^6^A-modified HCV RNAs are recruited by YTHDF proteins onto lipid droplets to impede the assembly of unmodified RNAs, thus inhibiting the production of HCV infectious particles. **(B)** m^6^A modification at the 5′ terminus of the pregenomic RNA (pgRNA) is required for HBV reverse transcription while m^6^A at the 3′ terminus of pgRNA lead to destabilization of HBV transcripts. **(C)** FTO promotes the demethylation of *RUNX1T1* pre-mRNA; this inhibits *SRSF2* binding and alters the alternative splicing of pre-mRNA, therefore promoting adipogenesis. **(D)** The expression of METTL3 is upregulated in type 2 diabetes. Upregulated METTL3 inhibits hepatic insulin sensitivity through m^6^A modification of Fasn mRNA and facilitates fatty acid metabolism.

Gokhale et al. outlined the mechanisms of m^6^A methylation in regulating the cellular activity of HCV in host cells (79). Knockdown of METTL3 and METTL14 with small interfering RNA (siRNA) increases the production of infectious virions of HCV, which has also been observed in YTHDF reader proteins (79). This may suggest that HCV replication can be inhibited by m^6^A modification. In addition, m^6^A can directly modulate the generation of HVC virions as revealed by immunofluorescent staining that YTHDF reader proteins are directly localized to the lipid droplets on which HCV virions assemble (Figure 3A) (79). Gokhale et al. discovered new signaling pathways affected by m^6^A modification after HCV infection, and reported that the infection alters m^6^A modification of cellular mRNA (80); for example, increased m^6^A levels of ROI kinase 3 (*RIOK3)* promotes its translation while decreased m^6^A levels of cold inducible RNA binding protein (*CIRBP*) promotes its alternative splicing (80). *RIOK3* may be translated into serine/threonine kinase, which may modulate antiviral pathways, and *CIRBR* encoding protein might bond RNA in the cellular stress response (88–90). Moreover, the innate immune response and endoplasmic reticulum (ER) stress can trigger this m^6^A methylation. However, it remains unclear whether the specific cellular changes involving those two mRNAs result from the innate immune response of the host to defend against infection or is normal cellular response to ensure robust replication of the HCV. Still, study of new m^6^A-modified transcripts could help better elucidate the dynamic changes that occur during viral infection and such materials may be potential antiviral-targets.

m^6^A modification is vital in the regulation of HBV activity (81). Although HBV is a double-stranded DNA (dsDNA) virus, the extension of its life (replication) is fulfilled by RNA transcripts (91). Imam et al. was the first time to identify m^6^A in HBV-infected cell lines and liver tissues of chronic HBV patients (Figure 3B) (81). They also reported the determinant m^6^A motif DRACH (D = A, G, or U and H = A, C, or U) within the epsilon loop near the 3′ terminus of HBV mRNA, and that the m^6^A site is located at A1907 (1905-1909, GGACA) (81). Knockdown of the m^6^A regulatory enzymes (YTHDF2, YTHDF3, METTL3, and METTL14) may increase the expression of HBV proteins (HBc and HBs), by reducing the stability of HBV RNA. However, m^6^A at the 5′ stem loop increases reverse transcription and silencing of MTEEL3 as well as METTL14 reduces reverse transcription (81). Thus, it is obvious that the effects of m^6^A in regulating HBV replication or infection is two-sided, which suggests that m^6^A modification in viral metabolism is more of a temporal and transient modulation to adapt to the environment of host cells.

## Potential Antiviral Therapies Targeting m^6^A and Future Prospects

Antiviral treatment is indispensable in HCC patients with viral hepatitis, not only for the inhibition of viral replication but also for the prevention of tumor progression (92, 93). The universal presence of m^6^A in various viruses makes it a potential target for a wide range of viral infection including HBV and HCV.

Interferons (IFNs) have been used in HCC patients with chronic viral hepatitis to impede virus infection and replication, which can effectively prevent the occurrence, recurrence and metastasis of HCC (94–96). Recent studies have shown that, one IFN-stimulated genes (ISG), ISG20, selectively recognize and promotes degradation of m^6^A-modified HBV transcripts (97). In that study, ISG20-mediated decay of specific HBV RNAs was inhibited by the knockdown of YTHDF2 reader proteins or methyltransferases. Moreover, this ISG20-mediated RNA decay occurred only at m^6^A sites of A1907 and combined with YTHDF2 reader protein to form a complex (97). These results lead to the assumption that any degradation process of virus transcripts can be regulated by the combination of YTHDF2 protein and its recognition at the epsilon stem loop (A1907), therefore opening a new gate for the treatment of HCC patients infected with hepatitis virus. There is evidence that 3-deazaadenosine (DAA), the S-adenosylhomocysteine (SAC) hydrolase inhibitor, could serve as a broad-spectrum antiviral (98). The inhibition of SAC hydrolase results in the accumulation of SAC, therefore impairing the generation of S-adenosylmethionine (SAM), from which METTL3 acquire methyl groups for m^6^A methylation (98). In several studies, DAA has been found to inhibit the replication of diverse viruses by indirectly targeting m^6^A modification, and the compound has demonstrated no detectable toxicity (99–102). After more *in vivo* validation, DAA could be a promising medicine for the treatment of viral hepatitis and the prevention of HCC.

In addition to currently available medicines that inhibit viral activity through m^6^A mechanisms, there may be a relationship between specific structural overlaps of viral genome and consensus sequences of m^6^A modification (103). The guanine-based putative-quadruplex-forming sequences (PQSs) is a conserved secondary structure in viral RNA, which can fold into guanine-quadruplexes (G4s) to regulate viral replication and inhibit protein translation in flaviviruses (79, 104, 105). If viruses can transform PQSs into G4s to counteract surveillance by host cells, G4s could be a potential antiviral target (106, 107). In one study, during the matching of viral PQS and m^6^A sequencing data, it was found that some sites of m^6^A occur within the loops of PQSs (103). In addition, mutation data have confirmed some overlapped m^6^A site are vital for HBV replication. Therefore, it is reasonable to propose that the colocalization of m^6^A and PQSs in HBV RNA might result from the topological control of m^6^A methylation, which could generate synergistic effects if it becomes a therapeutic target. However, more sequencing data of m^6^A is needed for further validation.

## Role of m^6^A Modification in Non-alcohol Fatty Liver Disease

m^6^A may play an important role in the metabolism of adipose tissues (85). Significant differences in m^6^A methylation landscape have been observed between NAFLD mice induced through a high-fat diet and normal mice (85). In addition, differential m^6^A genes are highly expressed in pathways associated with lipid metabolism (85). FTO has been widely studied since its relationship with obesity was discovered (35). Zhao et al. first demonstrated that demethylase FTO promotes the process of adipogenesis by reversing m^6^A modification (87). Silence of FTO can promote m^6^A modification, which strengthens the binding capacity of serine and arginine rich splicing factor 2 (*SRSF2*) with targeted exon Runt-related transcription factor 1 (*RUNX1T1*) (Figure 3C). This alters the alternative splicing of adipogenesis regulatory factor *RUNX1T1*, thereby slowing the differentiation of preadipocytes and adipogenesis (87). This positive regulation of FTO in lipogenesis has also been confirmed in other cellular networks (108, 109). FTO knockdown lowers the expression of cellular cycle regulators cyclin A2 (*CCNA2*) and cyclin-dependent kinase 2 (*CDK2*) but increases their m^6^A modification (108). In the meantime, their m^6^A-modified mRNAs are recognized and degraded by YTHDF2, resulting in suppressed adipogenesis. In addition, both FTO knockdown and METTL3 overexpression can decrease the accumulation of lipids by increasing mRNA m^6^A methylation (109). There are many other studies on the potential role of FTO in NAFLD, which we do not describe in detail here, because their mechanisms may not involve m^6^A modification or the methods used for m^6^A examination are not specific (110–112). In general, FTO and its related signaling could be a prospective research target in the treatment of NAFLD, and the prevention of NAFLD-related HCC.

Another major mechanism of NAFLD progression is insulin resistance (Figure 3D) (86). Intracellular METTL3 and m^6^A methylation in type 2 diabetes patients are positively related with insulin resistance (HOMA-β) and negatively related with β-cell function (84). METTL3 knockout in the liver of mice inhibits m^6^A modification and decreases the intracellular level of fatty acid synthase (*Fasn*). However, the depletion of METTL3 improves insulin sensitivity and inhibits fatty acid synthesis (84). These results indicate that insulin resistance is mediated by METTL3 through m^6^A modification of *Fasn* mRNA and it may accelerate the metabolism of fatty acids. Interestingly, another study reported that depletion of METTL14 in β cells in mice fed a high-fat diet led to seemingly paradoxical effects: insulin sensitivity increased while insulin secretion decreased (83). In that study, knockout of METTL14 lowered insulin secretion, increased insulin sensitivity, inhibited lipogenesis and enhanced lipolysis. Insulin sensitivity was enhanced due to the activation of AKT signaling and decreased gluconeogenesis; we speculate that the decrease in lipid accumulation might have resulted from the enhanced insulin signaling in the liver (83). All in all, METTL14 in β cells is positively correlated with lipogenesis and insulin secretion but negatively related with insulin sensitivity. Its function in insulin resistance and lipo-metabolism needs further validation in hepatocytes. However, METTL14 has great potential in the treatment of NAFLD, especially regarding to its multiple effects on insulin and lipid metabolism.

## Potential Therapies in Non-alcohol Fatty Liver Disease and Future Prospects

Although the number of people diagnosed with NAFLD is large, only a minority will fall into progressive liver disease or liver cancers (113). Thus, it is important to identify patients who are more likely to progress to HCC and conduct timely interventions.

The regulators of m^6^A methylation including METTL3, METTL14, YTHDF2, and FTO are potential targets based on their significant roles in the pathogenesis of NAFLD. For instance, dietary branched-chain amino acids (BCAA) can effectively regulate lipid metabolism in weanling piglets via m^6^A modification (114). In one study, high- and low-dose feeding of BCAA was associated with significantly lower levels of fatty acid synthesis compared to normal-dose feeding, due to the upregulation of lipolysis genes and downregulation of lipogenic genes. Moreover, a lower level of m^6^A methylation was identified with downregulation of METTL3 and FTO in the livers of the high-BCAA group (114). The results indicated that a dietary with high BCAAs can decrease lipid accumulation through the regulation of m^6^A modification.

The protective effects of betaine (trimethylglycine) on liver, including alleviation of impaired liver function and decreased lipid accumulation, have long been recognized (115, 116). Recently, it was confirmed that these protective effects are mediated by FTO through m^6^A modification (117). Betaine promotes lipolysis and lipid oxidative by reversing the hypomethylated-mRNA and overexpressed FTO in adipocytes of mice fed with high fat diet (118). In addition, this reversion requires the existence of AMP-activated protein kinase α1 subunit (*AMPK*α1) (118). Another study reported that lipopolysaccharide (LPS)-induced liver function and lipid metabolism disorder can be alleviated by curcumin through the upregulation of m^6^A (119). Furthermore, curcumin supplementation affected the expression of major m^6^A regulators, indicating its powerful hepatoprotective effect (119). There are plenty of other compounds such as exenatide and entacapone that directly target m^6^A regulator, that worth more researching in the future (120, 121).

## Conclusion

In summary, m^6^A modification plays an important role in the occurrence and development of HCC. Viral hepatitis and NAFLD are major risk factors for HCC, which shows a significant relationship with m^6^A methylation. In HCV and HBV, m^6^A regulates the infection and replication of virus. In NAFLD, it participates in insulin resistance and the accumulation of lipids. Therapeutic experiments regarding m^6^A have been conducted on two diseases, which will greatly promote the prevention of HCC. However, the chronic inflammation that accompanies these ailments will lead to liver fibrosis and/or cirrhosis, whose pathogeneses regarding m^6^A warrant further study. In HCC, m^6^A regulators specifically regulate mRNA splicing, translation, and degradation, while ncRNAs interact with m^6^A, leading to changes in gene expression; consequently, mRNAs and ncRNAs both regulate the progression of HCC. Therefore, in terms of the diversity of m^6^A in HCC, m^6^A-related molecules are potential biomarkers for early diagnosis and prospective therapeutic targets for clinical treatment. Furthermore, in the age of precision medicine, m^6^A-related therapies could be tailored based on the etiology and pathogenesis of HCC patients, which will optimize treatments and their benefits.

## Author Contributions

JL wrote the first draft of the manuscript. JQ conceived the idea of the manuscript. LZ and SY designed the structure of the manuscript. SZ and WZ revised, read, and approved the submitted version. All authors contributed to the article and approved the submitted version.

## Conflict of Interest

The authors declare that the research was conducted in the absence of any commercial or financial relationships that could be construed as a potential conflict of interest. The reviewer XH declared a shared affiliation, with no collaboration, with the authors to the handling editor at the time of review.

## Abbreviations

HCC: Hepatocellular carcinoma
NAFLD: Non-alcohol fatty liver disease
HBV: Hepatitis B virus
HCV: Hepatitis C virus
PD-L1: Programmed cell death ligand-1
m6A: N6-methyladenosine
mRNAs: Messenger RNAs
ncRNAs: Noncoding RNAs
METTL3: Methyltransferase-like 3
METTL14: Methyltransferase-like 14
WTAP: Wilm's tumor 1-associated protein
ZC3H13: Zinc finger CCCH domain-containing protein 13
ALKBH5: AlkB homolog 5
FTO: Fat mass and obesity-associated protein
YTHDC1: YTH domain-containing 1
YTHDF2: YTH N6-methyladenosine RNA-binding protein
RBM15: RNA-binding motif protein 15
HNRNPC: Heterogeneous nuclear ribonucleoprotein C
RBM15: RNA-binding motif protein 15
siRNA: Small interfering RNA
RIOK3: ROI kinase 3
CIRBP: Cold inducible RNA binding protein
dsDNA: Double-stranded DNA
IFNs: Interferons
ISGs: IFN-stimulated genes
DAA: 3-deazaadenosine
SAC: S-adenosylhomocysteine
SAM: S-adenosylmethionine
PQSs: Putative quadruplex-forming sequences
G4s: Guanine-quadruplexes
SNP: Single nucleotide polymorphism
NASH: Non-alcoholic steatohepatitis
SRSF2: Serine and arginine rich splicing factor 2
RUNX1T1: Runt-related transcription factor 1
CCNA2: Cellular cycle regulators cyclin A2
CDK2: Cyclin-dependent kinase 2
Fasn: Fatty acid synthase
BCAA: Branched-chain amino acids
AMPKα1: AMP-activated protein kinase α1 subunit
LPS: Lipopolysaccharide
ZCCHC4: Zinc finger CCHC-type containing 4
IL11: interleukin 11
SERPINE2: Serpin family E member 2
HIF-2α: Hypoxia-inducible factor 2α
SOCS2: Suppressor of cytokine signaling 2
ETS1: ETS proto-oncogene 1
HuR: Hu-antigen R
IGF2BP1: Insulin-like growth factor 2 mRNA-binding proteins
SRF: Serum response factor
ID2: DNA binding 2
PKM2: Pyruvate kinase M1/2
Me-RIP-PCR: Methylated- RNA immunoprecipitation- polymerase chain reaction
lincRNAs: Long noncoding RNAs
miRNAs: MicroRNAs
CircRNAs: circular RNAs
pri-miRNA: primary miRNA
DGCR8: DiGeorge syndrome chromosomal region 8
GATA3: GATA binding protein 3
pre-mRNA: Precursor mRNA
YAP: Yes-associated protein
CeRNA: Competitive endogenous RNA
PDX: Patient derived xenograft
PLGA-PEG NP: PEGylated PLGA nanoplatform
rRNAs: Ribosomal RNAs
CSAD: Cysteine sulfinic acid decarboxylase
GOT2: Glutamic-oxaloacetic transaminase 2
LLPS: Liquid-liquid phase separation
H3K36me3: H3 trimethylation at Lys36
carRNA: Chromosome-associated regulatory RNA
EMT: Epithelial mesenchymal transition
GC: Gastric cancer
ZMYM1: Zinc finger MYM-type containing 1
CRC: Colorectal cancer
NSCLC: Non-small cell lung cancer
AML: Acute myeloid leukemia.
